# Molecular data and species diagnosis in *Essigella* Del Guercio, 1909 (Sternorrhyncha, Aphididae, Lachninae)

**DOI:** 10.3897/zookeys.765.24144

**Published:** 2018-06-07

**Authors:** Thomas Théry, Mariusz Kanturski, Colin Favret

**Affiliations:** 1 University of Montreal, Department of Biological Sciences, Biodiversity Centre, 4101 E. Sherbrooke Street, Montreal QC, H1X 2B2 Canada; 2 Department of Zoology, Faculty of Biology and Environmental Protection, University of Silesia in Katowice, Bankowa 9, 40-007 Katowice, Poland

**Keywords:** Cryptic species, DNA sequences, Hemiptera, taxonomy

## Abstract

Morphological and molecular data are used to describe three new species of *Essigella* (Sternorrhyncha: Aphididae: Lachninae): *Essigella domenechi* **sp. n.**, *Essigella gagnonae* **sp. n.**, and *Essigella sorenseni* **sp. n.**; and to re-establish as valid *Essigella patchae* Hottes, 1957, **stat. n.**, until now considered a synonym of *E. pini* Wilson, 1919. The catalogue of *Essigella* species is updated. This study highlights the need and utility to use discreet DNA characters in aphid species diagnoses.

## Introduction

Morphological characters remain the commonest way to separate animal species, and they are conspicuously used in diagnoses and descriptions of new taxa. However, in the case of cryptic species, no or few morphological differences are available, and other kinds of taxon-related attributes must be employed as valuable diagnostic characters. DNA sequences permit the discovery of cryptic species and are used to separate them from their relatives (Hebert et al. 2003a, b, Cœur d’acier et al. 2014, Lukhtanov and Dantchenkoet 2017, Morinière et al. 2017). However, despite their reliability, they are seldom used specifically in diagnoses of new species, notably because they are not specifically recommended in the International Code of Zoological Nomenclature (Renner 2016).

*Essigella* (Sternorrhyncha: Aphididae: Lachninae) is an aphid genus found on the needles of various pinaceous hosts. Most species feed on true pines, *Pinus* Linnaeus, but *E. wilsoni* Hottes, 1957, is found only on Douglas firs, *Pseudotsuga* Carrière. *Essigella alyeska* Sorensen, 1988 is recorded on spruce, *Picea* A. Dietrich, although its typical host is *Pinus banksiana* Lamb. (Sorensen 1994). Most species of *Essigella* are considered monophagous except *E. californica* (Essig, 1909) and *E. pini* Wilson, 1919 which are oligophagous on *Pinus* (Sorensen 1994). Although all species are Nearctic in origin, *E. californica* was accidently introduced in several countries around the world (Théry et al. 2017). *Essigella* currently encompasses 15 valid taxa, with an additional 13 synonyms (Wilson 1919, Gillette and Palmer 1924, Hottes 1957, 1958, Sorensen 1988, 1994). Species are variable and show few diagnostic characters (Sorensen 1994). The genus was revised by Sorensen (1994) using morphometric data and multivariate analyses. Besides the 15 taxa he recognized, Sorensen (1994) notably divided *Essigella* into three subgenera: *Archeoessigella*, *Essigella* and *Lambersella*, two species series, and three species complexes. A recent molecular phylogenetic study did not support the validity of the three subgenera and of one of the species series (Théry et al. in press). Moreover, the phylogenetic results, combined with molecular species delimitation methods, revealed that two species, *Essigella californica* and *E. pini*, actually encompass four and two species, respectively. In the case of *E. pini*, one of the two species is suspected to be *E. patchae* Hottes, 1957, considered a synonym of *E. pini* by Sorensen (1994). Examination of type material of *E. californica* and *E. pini*, as well as that of their respective synonyms and reference specimens, indicates that the three cryptic species found within *E. californica* are new to science and confirms the validity of *E. patchae*.

In the present work, we describe as new the three cryptic species revealed by Théry et al. (in press): *Essigella domenechi* sp. n., *E. gagnonae* sp. n. and *E. sorenseni* sp. n. In addition, we re-establish *E. patchae* stat. n. and provide diagnostic characters to separate it and *E. pini*. Because these four species are difficult to distinguish morphologically, discreet DNA sequence data supplement classical morphological characters in the diagnoses.

## Materials and methods

### Abbreviations used

**CTT** Private Collection of T. Théry, Fleury les Aubrais, France;

**EMEC** Essig Museum of Entomology, University of California, Berkeley, CA, USA;

**QMOR** Ouellet-Robert Entomological Collection, University of Montreal, QC, Canada;

**UMSP** University of Minnesota Insect Collection, St Paul, MN, USA;

**USNM** National Aphid Collection, National Museum of Natural History, Beltsville, MD, USA.

### Taxon sampling

All *Essigella* specimens published here were collected recently in the USA and Canada (TT and CF), or are found in the Sorensen Collection at EMEC. Specimens studied were mainly viviparous apterae. Some viviparous alatae also were studied in the case of *E. patchae* for which the holotype is an alate. Recently collected specimens were preserved in 95% ethanol after collecting and subsequently kept at -20 °C or -80 °C. DNA extraction of at least one specimen per population was realized. It was non-destructive (Favret 2005), permitting us to keep the specimen as voucher. Those specimens were identified using the keys of Sorensen (1994) and Blackman and Eastop (2017). We compared our material with the type specimens of the valid species, *E. californica* (EMEC) and *E. pini* (UMSP), as well as those of their synonyms *E. claremontiana* Hottes, 1957, *E. cocheta* Hottes, 1957, *E. monelli* Hottes, 1957, *E. pineti* Hottes, 1957, *E. swaini* Hottes, 1957, for *E. californica*, and *E. patchae* for *E. pini* (EMEC, USNM). We also compared specimens of new taxa and of *E. patchae* with other *E. californica* specimens from the Sorensen Collection (EMEC), and of *E. pini* from UMSP and USNM.

### Preparation, measurements, and photographs

All new material was slide-mounted in Canada balsam and deposited in QMOR, CTT, and USNM, in the case of holotypes. Preparations were thick to reduce deformation due to compression. As far as possible, appendages were placed so that they be strictly horizontal permitting correct length and width measurements as well as to ascertain the correct location of dorsal and ventral setae of the hind femora and tibiae. Body length was measured from the frontal margin of the head to the posterior margin of the 7^th^ abdominal segment. The abdominal tergum being sclerotized with most segments fused, the cauda and 8^th^ segment sometimes telescope into the 7^th^, making standardized measurements difficult across specimens. Because of the likely deformation of the body due to a variable number of embryos, width measurements were taken only of the head, between the frontal interior margins of the compound eyes. Lengths of appendages were measured at their longest, including condyles, widths were measured at the widest part of the appendages. The length of the processus terminalis was taken from the distal margin of primary rhinarium to the apex of the antenna. The following abbreviations are applied:

**BL** body length;

**LAIII** length of third antennal segment;

**LAIV** length of fourth antennal segment;

**LAV** length of fifth antennal segment;

**LPRIV** length of primary rhinarium on fourth antennal segment;

**LPRV** length of primary rhinarium on fifth antennal segment;

**LPT** length of processus terminalis;

**HWE** head width at eyes;

**LURS** length of ultimate rostral segment;

**LMF** length of metafemur;

**WMF** width of metafemur;

**LMT** length of metatibia;

**WMT** width of metatibia;

**WS** width of siphunculus at external edges;

**LMB** length of metabasitarsus;

**LMD** length of metadistitarsus;

**LFS** length of longest frontal seta;

**LDMFS** length of longest dorsal metafemoral seta;

**LVMFS** length of longest ventral metafemoral seta;

**LDMTS** length of longest dorsal metatibial seta;

**LVMTS** length of longest ventral metatibial seta.

Entire non-prepared specimens were photographed with a Carl Zeiss Discovery.V20 stereoscope using an AxioCam HRc camera and a Zen 2012 Carl Zeiss Software, version 1.1.1.0. Pictures of slide-mounted specimens were realized using light microscope Nikon Eclipse E600 with differential interference contrast (DIC) and photographed by Nikon DS-Fi camera. Scanning electron microscope (SEM) photos were taken at the University of Silesia in Katowice (Poland) using a Hitachi SU8010 Field Emission Scanning Electron Microscope (FE-SEM) (Hitachi High-Technologies Corporation, Tokyo, Japan) at 5, 10 and 15 kV accelerating voltage with a secondary electron detector (ESD). For specimen preparation for SEM pictures, we followed the protocol of Kanturski et al. (2015). Measurements in diagnoses and descriptions are given in microns (µm) with standard deviation.

### Molecular data

The three new species were primarily revealed in the study of Théry et al. (in press) using DNA sequences of the genomes of the mitochondrion (*ATP6*, *COI*) and the obligate bacterial endosymbiont *Buchnera aphidicola* (*Gnd*) within populations of *E. californica* sensu lato. Indeed, *ATP6* and *Gnd* show similar properties as *COI* in species discrimination in barcoding (Hebert et al. 2003a, b, Chen et al. 2013, Lee et al. 2014). Sequence lengths were 663 base pairs (bp), 658 bp and 749 bp for *ATP6*, *COI* and *Gnd*, respectively (see Théry et al. in press, for GenBank accession numbers and other details).

## Taxonomy

The following species, including *E. patchae*, belong to the *E. californica* species complex, which also includes *E. hoerneri* Gillette & Palmer, 1924 (Sorensen 1994) (see discussion). All of these species, as well as *E. pini*, exhibit six dorsal setae at their 3^rd^ and 4^th^ abdominal segments (Sorensen 1994). However, this character is homoplastic within *Essigella* as *E. pini* and the *E. californica* complex are not closely related (Théry et al. in press); it is used here to distinguish the species of the *E. californica* complex and *E. pini* from the other species of the genus. Morphological and ecological (host plant identity) comparisons of specimens of the new species with type material of synonym species of *E californica* and *E. hoerneri* permitted to reject the possibility that our new species correspond to one of those synonyms.

### Essigella domenechi sp. n.

Taxon classificationAnimaliaHemipteraAphididae

http://zoobank.org/390343A7-D620-4578-93A8-A5BBBF7FE00F

Figure 1d

#### Holotype.

viviparous aptera, USA, California, Alpine Co., 38.328°N 119.637°W, 10.vii.2013, on *Pinus albicaulis*, T. Théry & C. Favret *leg.* (USNM). **Paratypes.** 8 viviparous apterae, same data as holotype (QMOR, CTT).

**Figure 1.**
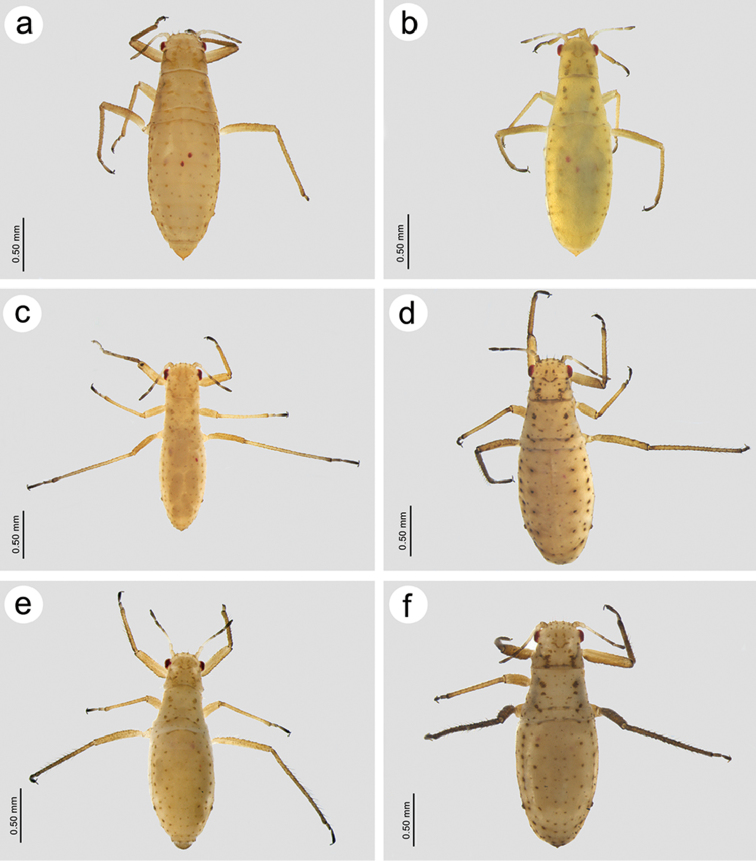
Habitus of viviparous apterae of **a** *Essigella pini* **b** *E. patchae* **c** *E. californica* **d** *E. domenechi* sp. n. **e** *E. gagnonae* sp. n. **f**. *E. sorenseni* sp. n.

#### Diagnosis.

**Figure 2.**
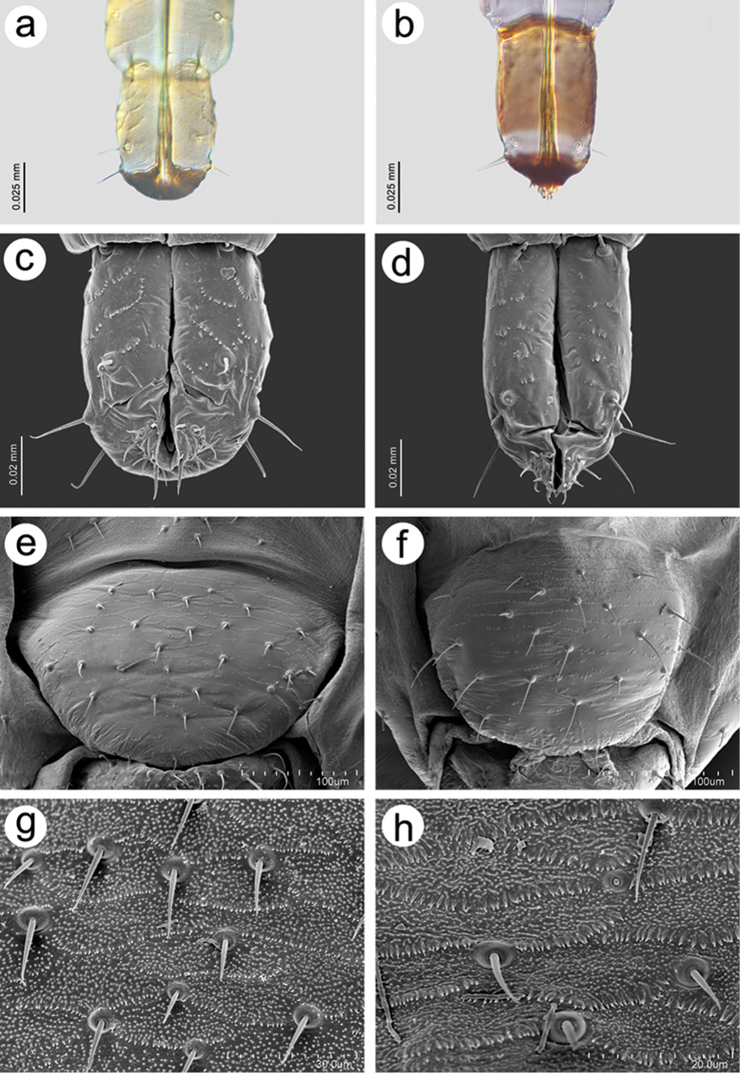
Morphological structures in *Essigella pini* and in *E. patchae*: **a** URS in *E. pini* (slide-mounted specimen) **b** URS in *E. patchae* (slide-mounted specimen) **c** URS in *E. pini* (SEM) **d** URS in *E. patchae* (SEM) **e** genital plate in *E. pini* (SEM) **f** genital plate in *E. patchae* (SEM) **g** details in genital plate in *E. pini* (SEM) **h** details in genital plate in *E. patchae* (SEM).

Like species of the *E. californica* complex and *E. pini*, *E. domenechi* sp. n. has its 3^rd^ and 4^th^ abdominal dorsal terga usually bearing six setae. The species can be distinguished from *E. patchae* by the presence of rows of spinules on the URS (absent or faint in *E. patchae*; Fig. 2b, d); from *E. pini* by a relatively elongate URS with subparallel lateral margins (URS with margins rounded and convergent at base in *E. pini*; Fig. 2a, c); from *E. gagnonae* sp. n. and *E. sorenseni* sp. n. with the following characters: tibiae and femora more or less concolorous showing almost or same color as that of body (pro- and metatibiae and metafemora conspicuously darkened in *E. sorenseni* sp. n., pro- and metatibiae sometimes slightly darkened in *E. gagnonae* sp. n.), dorsal tegument thick; width of head between eyes = 300.7 ± 14.2 (289.0 ± 13.3 for *E. gagnonae* sp. n., and 353.6 ± 15.3 for *E. sorenseni* sp. n.); ratio of 3^rd^ / 5^th^ antennal segments < 1.6 (< 1.6 for *E. gagnonae* sp. n. but > 1.6 in *E. sorenseni* sp. n.); overall pubescence short or medium-sized with average length of the longest dorsal seta of metafemora = 29.7 ± 4.2 (59.8 ± 9.8 for *E. gagnonae* sp. n., and 51.2 ± 10.7 for *E. sorenseni* sp. n.; average length of the longest ventral seta of metafemora = 32.6 ± 4.5 (43.1 ± 5.4 for *E. gagnonae* sp. n., and 54.4 ± 5.6 for *E. sorenseni* sp. n.); average length of the longest dorsal seta of metatibiae = 44.0 ± 8.1 (85.7 ± 10.8 for *E. gagnonae* sp. n., and 76.4 ± 15.8 for *E. sorenseni* sp. n.); average length of the longest ventral seta of metatibiae = 37.5 ± 7.0 (49.4 ± 9.5 for *E. gagnonae* sp. n., and 67.7 ± 12.0 for *E. sorenseni* sp. n.); average length of the longest frontal seta = 32.6 ± 7.5 (58.7 ± 8.3 for *E. gagnonae* sp. n., and 53.4 ± 11.9 for *E. sorenseni* sp. n.); average number of setae of the genital plate = 22.0 ± 2.1 (23.6 ± 2.1 for *E. gagnonae* sp. n., and 31.6 ± 1.7 for *E. sorenseni* sp. n.). *Essigella domenechi* sp. n. is morphologically not distinguishable from *E. californica*, the latter being highly variable, nor from *E. hoerneri*. *Essigella domenechi* sp. n. can be separated from *E. californica*, *E. gagnonae* sp. n., *E. hoerneri* and *E. sorenseni* sp. n. with the DNA characters shown in Table 1.

**Table 1.** Diagnostic nucleotide differences between *E. domenechi* sp. n. and *E. californica*, *E. gagnonae* sp. n., *E. hoerneri*, and *E. sorenseni* sp. n. for *ATP6*, *COI*, and *Gnd*.

Gene	*ATP6* (663 bp)				*COI* (658 bp)								*Gnd* (749 bp)	
Site	4	71	227	324	190	229	334	386	418	565	619	625	219	621
*E. domenechi* sp. n.	**C**	**C**	**C**	**G**	**G**	**G**	**A**	**G**	**C**	**G**	**G**	**G**	**C**	**C**
*E. gagnonae* sp. n.	T	T	T	A	A	A	T	A	T	A	A	A	A	A
*E. sorenseni* sp. n.	T	T	T	A	A	A	T	A	T	A	A	A	A	A
*E. californica*	T	T	T	A	A	A	T	A	T	A	A	A	A	A
*E. hoerneri*	T	T	T	A	A	A	T	A	T	A	A	A	A	A

#### Description.

**Viviparous apterae** (prepared specimens): body with pale tegument, with visible pigmented scleroites; dorsal tegument visibly thicker, sclerotized. Legs quite pale, concolorous, more or less the same color than that of body. Antennae pale, the 5^th^, the 4^th^ and the apical third part of the 3^rd^ segment of antennae darkened. URS elongated, with lateral margins subparallel, bearing rows of spinules. Overall pubescence short to medium-sized, dorsal setae of appendages incrassate, ventral ones acute. Terga of abdominal segments 3 and 4 with six dorsal setae. Genital plate with 19–25 setae (22.0 ± 2.1) (n = 6). Cauda obvious but not too protruding, apically rounded, slightly turned upward. BL: 1600–2100 (1800 ± 170) (n = 7). HWE: 283.2–326.0 (300.7 ± 14.2) (n = 7), LAIII: 162.2–184.6 (171.8 ± 6.5) (n = 13), LAIV: 96.4–106.7 (101.0 ± 4.1) (n = 9), LAV: 113.7–124.4 (120.2 ± 4.1) (n = 5), LPRIV: 20.8–25.8 (23.0 ± 1.6) (n = 9), LPRV: 17.6–21.9 (19.6 ± 1.4) (n = 9), LPT: 8.5–14.5 (11.9 ± 1.9) (n = 9), LURS: 71.4–79.2 (75.2 ± 2.8) (n = 6), LMF: 675.7–728.8 (708.3 ± 24.2) (n = 6), WMF: 68.2–77.3 (74.9 ± 2.9) (n = 11), LMT: 975.1–1074.4 (1027.8 ± 38.2) (n = 9), WMT: 36.8–43.9 (41.4 ± 2.2) (n = 12), WS: 36.7–43.5 (40.4 ± 2.2) (n = 9), LMB: 107.2–114.4 (110.9 ± 2.3) (n = 11), LMD: 189.4–206.4 (194.9 ± 6.9) (n = 11), LFS: 18.7–39.5 (32.6 ± 7.5) (n = 7), LDMFS: 25.2–36.8 (29.7 ± 4.2) (n = 12), LVMFS: 26.1–44.0 (32.6 ± 4.5) (n = 12), LDMTS: 33.7–61.8 (44.0 ± 8.1) (n = 12), LVMTS: 24.7–48.1 (37.5 ± 7.0) (n = 12).

#### Comments.

USA, California, on *Pinus albicaulis* Engelmann, known from Stanislaus National Forest at high elevation (type series). The species probably occurs in other high mountains where *P. albicaulis* is present. This species corresponds to the *E. californica* population living on *P. albicaulis* (cluster H3) shown in Théry et al. (in press).

#### Etymology.

This species is dedicated to Boris Domenech, PhD student at the University of Montreal (QC, Canada) for his comments in genetic analyses with which the species was discovered.

### Essigella gagnonae sp. n.

Taxon classificationAnimaliaHemipteraAphididae

http://zoobank.org/53A36CBB-AE8E-42FD-B792-C431EE48BBD4

Figs 1e , 3b

#### Holotype.

viviparous aptera, USA, Nevada, Douglas Co., 38.999°N 119.896°W, 10.vii.2013, on *Pinus monticola*, T. Théry & C. Favret *leg.* (USNM). **Paratypes.** 1 viviparous aptera, same data as holotype (QMOR); 12 viviparous apterae, California, El Dorado Co., 38.834°N 120.042°W, 09.vii.2013, on *Pinus monticola*, T. Théry & C. Favret *leg.*, specimens on 10 slides (QMOR, CTT); 5 viviparous apterae, California, Lassen Co., HWY 89, 6 km N Jct HWY 36 & 89, 6600’, S of Lassen Nat’l Park (77G20), 10.vii.1977, on *Pinus monticola*, J. T. Sorensen *leg.*, specimens on 1 slide (EMEC); 5 viviparous apterae, Californica, Alpine Co., E side Ebbett’s Pass, HWY 4, 3 km E summit (77G41), 17.vii.1977, on *Pinus monticola*, J. T. Sorensen *leg.*, specimens on 1 slide (EMEC); 13 viviparous apterae, Washington, Kitsap Co., 8 km S Hood Canal Bridge, HWY 3 (78G49), 09.vii.1978, on *Pinus monticola*, J. T. Sorensen *leg.*, specimens on 3 slides (4 + 4 + 5) (EMEC); 8 viviparous apterae, Washington, Grays Harbor Co., 16 km W Amanda Park, HWY 101 (78G54), 10.vii.1978, on *Pinus monticola*, J. T. Sorensen leg., specimens on 2 slides (4 + 4) (EMEC); 5 viviparous apterae, Nevada, Washoe Co., Mt Rose, Summit, Cmpgd, Toiyabe Nat’l Forest (78H9), 02.viii.1978, on *Pinus monticola*, J. T. Sorensen *leg.*, specimens on 2 slides (2 + 3) (EMEC).

#### Diagnosis.

Like species of the *E. californica* complex and *E. pini*, *E. gagnonae* sp. n. has its 3^rd^ and 4^th^ abdominal dorsal terga usually bearing six setae. It can be distinguished from *E. patchae* by the presence of spinules on the URS (absent or faint in *E. patchae*; Fig. 2b, d); from *E. pini* by a relatively elongate URS with subparallel lateral margins (URS with margins rounded and convergent at base in *E. pini*; Fig. 2a, c); from *E. domenechi* sp. n. and *E. sorenseni* sp. n. with the following characters: legs ranging from concolorous and slightly darker than body, to pro- and metatibiae slightly darkened with mesotibiae lighter and metafemora pale (tibiae concolorous in *E. domenechi* sp. n., pro- and metatibiae, and metafemora conspicuously darkened in *E. sorenseni* sp. n.); width of head between eyes = 289.0 ± 13.3 (300.7 ± 14.2 for *E. domenechi* sp. n., and 353.6 ± 15.3 for *E. sorenseni* sp. n.); ratio of 3^rd^ / 5^th^ antennal segments < 1.6 (< 1.6 for *E. domenechi* sp. n. but > 1.6 in *E. sorenseni* sp. n.); overall pubescence medium-sized to long with average length of the longest dorsal setae of metafemora = 59.8 ± 9.8 (29.7 ± 4.2 for *E. domenechi* sp. n., and 51.2 ± 10.7 for *E. sorenseni* sp. n.); average length of the longest ventral seta of metafemora = 43.1 ± 5.4 (32.6 ± 4.5 for *E. domenechi* sp. n., and 54.4 ± 5.6 for *E. sorenseni* sp. n.); average length of the longest dorsal seta of metatibiae = 85.7 ± 10.8 (44.0 ± 8.1 for *E. domenechi* sp. n., and 76.4 ± 15.8 for *E. sorenseni* sp. n.); average length of the longest ventral seta of metatibiae = 49.4 ± 9.5 (37.5 ± 7.0 for *E. domenechi* sp. n., and 67.7 ± 12.0 for *E. sorenseni* sp. n.); average length of the longest frontal seta = 58.7 ± 8.3 (32.6 ± 7.5 for *E. domenechi* sp. n., and 53.4 ± 11.9 for *E. sorenseni* sp. n.); average number of setae of the genital plate = 23.6 ± 2.1 (22.0 ± 2.1 for *E. domenechi* sp. n., and 31.6 ± 1.7 for *E. sorenseni* sp. n.). *Essigella gagnonae* sp. n. is for now morphologically not distinguishable from *E. californica*, the latter being highly variable, nor from *E. hoerneri*. *Essigella gagnonae* sp. n. can be separated from *E. californica*, *E. domenechi* sp. n., *E. hoerneri*, and *E. sorenseni* sp. n. with the DNA characters shown in Table 2.

#### Description.

**Figure 3.**
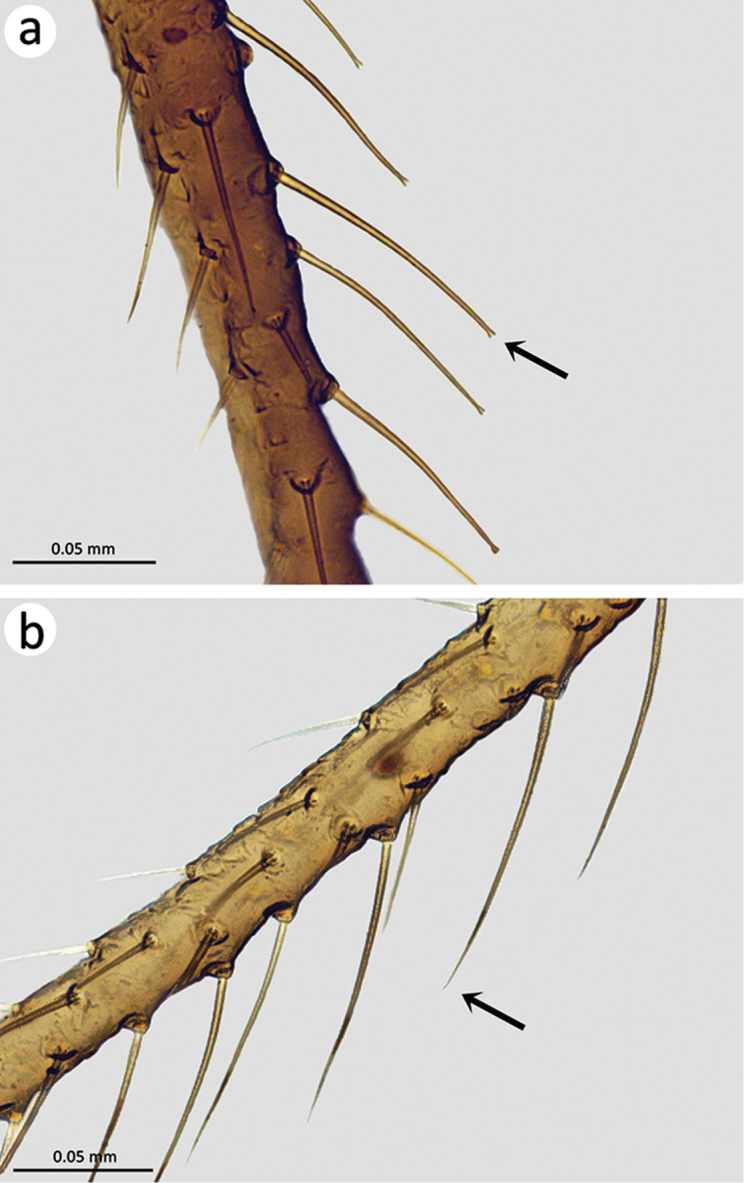
Details of dorsal setae of metatibia in **a** *E. sorenseni* sp. n. (slide-mounted specimen) **b** *E. gagnonae* sp. n. (slide-mounted specimen).

**Viviparous apterae** (prepared specimens): body with pale tegument sometimes slightly yellowish, with visible pigmented scleroites. Legs ranging from concolorous, slightly darker than body, to pro- and metatibiae slightly darkened, darker than body and mesotibiae. Antennae pale, the 5^th^, the 4^th^ and the apical third part of the 3^rd^ segment darkened. URS elongated, with lateral margins subparallel, bearing rows of spinules. Overall pubescence medium-sized to long, dorsal setae of appendages incrassate, ventral ones acute, in specimens with very long dorsal setae in metafemora and metatibiae (> 100 µm), these setae almost acute to acute (Fig. 3b), straight to sinuated. Terga of abdominal segments 3 and 4 with six dorsal setae. Genital plate with 21–26 setae (23.6 ± 2.1) (n = 9). Cauda obvious but not too protruding, apically rounded, slightly turned upward. BL: 1600–2000 (1800 ± 130) (n = 19). HWE: 271.0–311.9 (289.0 ± 13.3) (n = 13), LAIII: 157.6–197.4 (178.1 ± 11.1) (n = 29), LAIV: 90.2–111.6 (99.7 ± 6.3) (n = 33), LAV: 116.0–141.6 (125.4 ± 5.8) (n = 20), LPRIV: 21.5–29.1 (24.3 ± 1.8) (n = 21), LPRV: 18.5–22.6 (20.6 ± 1.2) (n = 18), LPT: 7.6–16.8 (12.1 ± 2.5) (n = 23), LURS: 64.5–79.8 (72.0 ± 3.8) (n = 18), LMF: 650.3–798.5 (707.3 ± 38.6) (n = 22), WMF: 69.5–104.6 (87.0 ± 10.8) (n = 29), LMT: 876.1–1104.2 (999,9 ± 67.4) (n = 25), WMT: 33.8–52.5 (42.3 ± 4.1) (n = 40), WS: 34.4–42.6 (38,9 ± 2.5) (n = 18), LMB: 101.8–131.0 (116.1 ± 8.0) (n = 36), LMD: 180.3–209.9 (195.0 ± 8.6) (n = 34), LFS: 44.4–80.2 (58.7 ± 8.3) (n = 26), LDMFS: 42.0–82.9 (59.8 ± 9.8) (n = 43), LVMFS: 31.5–52.6 (43.1 ± 5.4) (n = 42), LDMTS: 60.9- 107.7 (85.7 ± 10.8) (n = 46), LVMTS: 30.5–74.5 (49.4 ± 9.5) (n = 46).

#### Comments.

USA, California, Nevada, and Washington, on *Pinus monticola* Douglas ex D. Don. The species occurs in elevated places where *P. monticola* is present. This species corresponds to the *E. californica* population living on *P. monticola* (cluster H2) shown in Théry et al. (in press).

#### Etymology.

This species is dedicated to Édeline Gagnon, PhD student at the University of Montreal (QC, Canada) for her help in genetic analyses with which the species was discovered.

**Table 2.** Diagnostic nucleotide differences between *E. gagnonae* sp. n. and *E. californica*, *E. domenechi* sp. n., *E. hoerneri* and *E. sorenseni* sp. n. for *ATP6*, *COI*, and *Gnd*.

Gene	*ATP6* (663 bp)	*COI* (658 bp)			*Gnd* (749 bp)
Site	260	28	235	271	665
*E. gagnonae* sp. n.	**G**	**G**	**C**	**C**	**C**
*E. domenechi* sp. n.	A	A	T	A	T
*E. sorenseni* sp. n.	A	A	T	A	A
*E. californica*	A	A	T	A	A
*E. hoerneri*	A	A	T	A	T

### Essigella sorenseni sp. n.

Taxon classificationAnimaliaHemipteraAphididae

http://zoobank.org/4C35698B-A28C-4794-8AE8-C9A6BA84541F

Figs 1f , 3a

#### Holotype.

viviparous aptera, USA, California, Sonoma Co., 38.534°N 123.276°W, 02.vii.2013, on *Pinus muricata*, T. Théry & C. Favret *leg.* (QMOR). **Paratypes.** 14 viviparous apterae, same data than holotype, specimens on 14 slides (QMOR, CTT); 3 viviparous apterae, California, Mendocino Co., 38.984°N 123.696°W, 03.vii.2013, on *Pinus muricata*, T. Théry & C. Favret *leg.*, specimens on 3 slides (QMOR, CTT); 6 viviparous apterae, California, Mendocino Co., HWY 1, 5 km of Albion, Little River Road, 23.vii.1977, on *Pinus muricata*, 77G52, J. T. Sorensen *leg.*, specimens on 3 slides (2 + 2 + 2) EMEC); 13 viviparous apterae, California, Humbodlt Co., nr Little River State Beach, 17 km N Arcata, HWY 101, 04.vii.1978, on *Pinus muricata*, 78G3, J. T. Sorensen *leg.*, specimens on 4 slides (4 + 4 + 4 + 1) (EMEC).

#### Diagnosis.

Like species of the *E. californica* complex and *E. pini*, *E. sorenseni* sp. n. has its 3^rd^ and 4^th^ abdominal dorsal terga usually bearing six setae. It can be distinguished from *E. patchae* by the presence of spinules on the URS (absent or faint in *E. patchae*; Fig. 2b, d); from *E. pini* by a relatively elongate URS with subparallel lateral margins (URS with margins rounded and convergent at base in *E. pini*; Fig. 2a, c); from *E. domenechi* sp. n. and *E. gagnonae* sp. n. with the following characters: usually pro- and metatibiae conspicuously darkened with mesotibiae always lighter, metafemora darkened (tibiae concolorous, metafemora pale in *E. domenechi* sp. n., concolorous or with pro- and metatibiae slightly darkened with mesotibiae lighter, metafemora pale in *E. gagnonae* sp. n.); width of head between eyes = 353.6 ± 15.3 (300.7 ± 14.2 for *E. domenechi* sp. n., and 289.0 ± 13.3 for *E. gagnonae* sp. n.); ratio of 3^rd^ / 5^th^ antennal segments > 1.6 (< 1.6 for *E. domenechi* sp. n. and *E. gagnonae* sp. n.); overall pubescence medium-sized to long with average length of the longest dorsal setae of metafemora = 51.2 ± 10.7 (29.7 ± 4.2 for *E. domenechi* sp. n., and 59.8 ± 9.8 for *E. gagnonae* sp. n.); average length of the longest ventral seta of metafemora = 54.4 ± 5.6 (32.6 ± 4.5 for *E. domenechi* sp. n., and for 43.1 ± 5.4 for *E. gagnonae* sp. n.); average length of the longest dorsal seta of metatibiae = 76.4 ± 15.8 (44.0 ± 8.1 for *E. domenechi* sp. n., and 85.7 ± 10.8 for *E. gagnonae* sp. n.); average length of the longest ventral seta of metatibiae = 67.7 ± 12.0 (37.5 ± 7.0 for *E. domenechi* sp. n., and 49.4 ± 9.5 for *E. gagnonae* sp. n.); average length of the longest frontal setae = 53.4 ± 11.9 (32.6 ± 7.5 for *E. domenechi* sp. n., and for 58.7 ± 8.3 *E. gagnonae* sp. n.); average number of setae of the genital plate = 31.6 ± 1.7 (22.0 ± 2.1 for *E. domenechi* sp. n., and 23.6 ± 2.1 for *E. gagnonae* sp. n.). *Essigella sorenseni* sp. n. is for now morphologically not distinguishable from *E. californica*, the latter being highly variable, nor from *E. hoerneri*. *E. sorenseni* sp. n. can be separated from *E. californica*, *E. domenechi* sp. n., *E. gagnonae* sp. n., and *E. hoerneri* with the DNA characters shown in Table 3.

#### Description.

**Viviparous apterae** (prepared specimens): body with a yellowish tegument more or less darkened at joints depending on the specimens, with conspicuous and pigmented scleroites. Legs usually with pro- and metatibiae conspicuously darkened, much darker than body and mesotibiae. Antennae pale, the 5^th^, the 4^th^ and the apical third part of the 3^rd^ segment darkened. URS elongated, with lateral margins subparallel, bearing rows of spinules. Overall pubescence medium-sized to long, dorsal setae of appendages incrassate, ventral ones acute, in specimens with very long dorsal setae on metafemora and metatibiae (> 100 µm), these setae not acute or seemingly acute but still incrassate (Fig. 3a), the setae sometimes curved at base. Terga of abdominal segments 3 and 4 with six dorsal setae. Genital plate with 29–34 setae (31.6 ± 1.7) (n = 10). Cauda obvious but not protruding, apically rounded, slightly turned upward. BL: 1900–2300 (2200 ± 110) (n = 21). HWE: 322.3–376.1 (353,6 ± 15.3) (n = 17), LAIII: 207.5–256.3 (233.6 ± 12.8) (n = 25), LAIV: 98.3–130.0 (112.2 ± 7.1) (n = 34), LAV: 120.1–139.8 (127.9 ± 4.7) (n = 23), LPRIV: 19.9–27.8 (24.1 ± 1.8) (n = 28), LPRV: 17.4–23.4 (19.5 ± 1.5) (n = 21), LPT: 11.6–15.7 (13.8 ± 1.4) (n = 21), LURS: 74.1–86.4 (80.5 ± 3.2) (n = 21), LMF: 702.3–927.8 (810.8 ± 58.9) (n = 26), WMF: 87.5–128.9 (103.1 ± 11.3) (n = 36), LMT: 1064.2–1450.4 (1233.4 ± 95.1) (n = 26), WMT: 49.5–76.0 (55.1 ± 5.1) (n = 37), WS: 39.0–44.6 (41.4 ± 1.7) (n = 22), LMB: 118.5–140.3 (130.3 ± 6.4) (n = 38), LMD: 183.4–212.5 (198.1 ± 7.9) (n = 34), LFS: 31.9–82.7 (53.4 ± 11.9) (n = 25), LDMFS: 34.2–79.4 (51.2 ± 10.7) (n = 45), LVMFS: 43.4–66.0 (54.4 ± 5.6) (n = 44), LDMTS: 47.7–113.8 (76.4 ± 15.8) (n = 46), LVMTS: 45.9–92.2 (67.7 ± 12.0) (n = 45).

#### Comments.

USA, California, on *Pinus muricata* D. Don, known from Humboldt, Mendocino, and Sonoma counties (type series), but probably present everywhere on the coastal range in California where *P. muricata* occurs. This species corresponds to the *E. californica* population living on *P. muricata* (cluster H1) shown in Théry et al. (in press).

#### Etymology.

This species is dedicated to John T. Sorensen, aphid specialist who eminently revised the genus *Essigella* in 1994, for his advice and hospitality accorded to the authors (TT and CF) in California.

**Table 3.** Diagnostic nucleotide differences between *E. sorenseni* sp. n. and *E. californica*, *E. domenechi* sp. n., *E. gagnonae* sp. n., and *E. hoerneri* for *ATP6*, *COI*, and *Gnd*.

Gene	*ATP6* (663 bp)		*COI* (658 bp)	*Gnd* (749 bp)		
Site	110	399	247	198	407	431
*E. sorenseni* sp. n.	**C**	**C**	**T**	**T**	**C**	**G**
*E. domenechi* sp. n.	T	T	C	C	T	T
*E. gagnonae* sp. n.	T	T	C	C	T	T
*E. californica*	T	T	C	C	T	T
*E. hoerneri*	T	T	C	C	T	T

### Essigella patchae

Taxon classificationAnimaliaHemipteraAphididae

Hottes, 1957 stat. n.

Figs 1b , 2b, d, f, h

Essigella patchae Hottes, 1957: 98 (Type locality: “Stillwater, Maine”). Holotype viviparous alate in USNM. Sorensen 1994: 49 [synonymy with E. pini Wilson]. **Status re-established**.

#### Other examined material.

1 viviparous alate and 1 viviparous aptera, Canada, Québec, Saint-Hippolyte, N45.991 - W74.009, ix.2015, on *Pinus strobus*, C. Favret *leg.* (QMOR); 1 viviparous aptera, Saint-Hippolyte, N45.989 - W74.005, ix.2016, on *Pinus strobus*, T. Théry *leg.* (QMOR); 1 viviparous aptera, Saint-Hippolyte, N45.989 - W74.005, ix.2017, on *Pinus strobus*, T. Théry *leg.* (QMOR).

#### Diagnosis.

Like species of the *E. californica* complex and *E. pini*, *E. patchae* has its 3^rd^ and 4^th^ dorsal abdominal terga usually with six setae. *Essigella patchae* can be distinguished from the other species of the *E. californica* complex species and from *E. pini* by its ultimate rostral segment (URS) exhibiting no or barely visible rows of spinules (Fig. 2b, d), which are clearly visible in other species of the *E. californica* complex and also in *E. pini* (Fig. 2a, c). *Essigella patchae* can also be differentiated from *E. pini* by having the general shape of the URS more elongated with lateral margins almost parallel (Fig. 2b, d) (margins more rounded and convergent at base in *E. pini*; Fig. 2a, c); shorter cauda than that of *E. pini* which can be elongated and acute; genital plate with fewer setae (15–20 vs 26–30 in *E. pini*), longer in *E. patchae* in comparison with *E. pini* (Fig. 2e, f), with spinules of the genital plate tegument more developed in *E. patchae* (Fig. 2g, h).

#### Host plant and distribution.

The species is currently known from its type locality in Maine (USA) and from one locality in Quebec (Canada) on *Pinus strobus* Linnaeus (see discussion).

### Simplified key to species of the *Essigella californica* complex, for viviparous apterae

Due to the variability of preparation, notably cover slip-induced deformations, teneral specimens, and general morphological variability, several specimens and the identity of the host plant are required to best use this key.

**Table:** 

1	Dorsal terga 3 and 4 usually with six setae	***E. californica* complex, *E. pini***...**2**
–	Dorsal terga 3 and 4 usually with more than six setae	**other *Essigella* species** (see Sorensen 1994)
2	Western North American species	**3**
–	Eastern North American species	**7**
3	On pinyon pines (*Pinus cembroides*, *P. edulis*, *P. monophylla*, *P. quadrifolia*)	***E. hoerneri***
–	Not on pinyon pines	**4**
4	On *Pinus albicaulis*, *P. monticola*, or *P. muricata*	**5**
–	On other pine species	***E. californica***
5	Ratio of LAIII / LAV > 1.6 (1.66–1.94), number of setae on genital plate > 27 (29–34), on *P. muricata*	***E. sorenseni* sp. n.**
–	Ratio of LAIII / LAV < 1.6 (1.29–1.54), number of setae on genital plate < 27 (19–26)	**6**
6	Dorsal setae of metafemora (25.2–36.8 µm) and of metatibiae (33.7–61.8 µm) short, on *P. albicaulis*	***E. domenechi* sp. n.**
–	Dorsal setae of metafemora (42.0–82.9 µm) and of metatibiae (60.9–107.7 µm) long, on *P. monticola*	***E. gagnonae* sp. n.**
7	Ultimate Rostral Segment (URS) with rows of spinules; sides of URS convex, convergent at base (Fig. 2a, c), number of setae on genital plate > 24 (26–30)	***E. pini***
–	Ultimate Rostral Segment (URS) without or with barely visible rows of spinules (Fig. 2b, d); sides of URS subparallel, not convergent at base, number of setae on genital plate < 24 (15–20)	***E. patchae***

### Catalogue of *Essigella* species

Genus *Essigella* del Guercio, 1909: 329

Type species : *Lachnus californicus* Essig, 1909: 1

= *Archeoessigella* Sorensen, 1994: 21; [**new synonym**]

= *Lambersella* Sorensen, 1994: 29; [**new synonym**]

*Essigella alyeska* Sorensen, 1988: 118; Sorensen 1994: 72

*Essigella californica* (Essig), 1909: 1; Sorensen 1994: 53

= *Lachnus californicus* Essig, 1909: 1

= *Essigella claremontiana* Hottes, 1957: 79 [synonymy by Sorensen 1994: 53]

= *Essigella cocheta* Hottes, 1957: 82 [synonymy by Sorensen 1994: 53]

= *Essigella monelli* Hottes, 1957: 95 [synonymy by Sorensen 1994: 53]

= *Essigella pineti* Hottes, 1957: 101 [synonymy by Sorensen 1994: 53]

= *Essigella swaini* Hottes, 1957: 105 [synonymy by Sorensen 1994: 53]

*Essigella critchfieldi* Sorensen, 1994: 75

*Essigella domenechi* sp. n.

*Essigella eastopi* Sorensen, 1994: 30

*Essigella essigi* Hottes, 1957: 84; Sorensen 1994: 45

*Essigella fusca fusca* Gillette & Palmer, 1924: 6 ; Sorensen 1994: 34

= *Essigella fusca* Gillette & Palmer, 1924: 6

= *Essigella agilis* Hottes, 1957: 71 [synonymy by Sorensen 1994: 34]

= *Essigella palmerae* Hottes, 1957: 96 [synonymy by Sorensen 1994: 34]

*Essigella fusca voegtlini* Sorensen, 1994: 39

*Essigella gagnonae* sp. n.

*Essigella hillerislambersi* Sorensen, 1994: 41

*Essigella hoerneri* Gillette & Palmer, 1924: 5; Sorensen 1994: 62

= *Essigella gillettei* Hottes, 1957: 88 [synonymy by Sorensen 1994: 62]

= *Essigella maculata* Hottes, 1957: 93 [synonymy by Sorensen 1994: 62]

*Essigella kathleenae* Sorensen, 1988: 115; Sorensen 1994: 26

*Essigella kirki* Sorensen, 1988: 121; Sorensen 1994: 22

*Essigella knowltoni braggi* Hottes, 1957: 73; Sorensen 1994: 84

= *Essigella braggi* Hottes, 1957: 73 [new status by Sorensen 1994: 84]

= *Essigella robusta* Hottes, 1957: 103 [synonymy by Sorensen 1994: 84]

*Essigella knowltoni knowltoni* Hottes, 1957: 92 [new status by Sorensen 1994: 78]

= *Essigella knowltoni* Hottes, 1957: 92

*Essigella patchae* Hottes, 1957: 98; Sorensen 1994: 49; [stat. n.]

*Essigella pini* Wilson, 1919: 2; Sorensen 1994: 49

*Essigella sorenseni* sp. n.

*Essigella wilsoni* Hottes, 1957: 106; Sorensen 1994: 67

= *Essigella pergandei* Hottes, 1957: 100 [synonymy by Sorensen 1994: 67]

= *Essigella oregonensis* Hottes, 1958: 155 [synonymy by Sorensen 1994: 67]

## Discussion

### *Essigella californica*

Sorensen, in his revision of the genus *Essigella* (1994) had already documented the existence of different host-associated groups within *E. californica*. He notably mentioned populations living on *Pinus flexilis* E. James and *P. lambertiana* Douglas, populations that he nevertheless considered as exhibiting intraspecific variation (Sorensen 1983, 1994). Populations from those two pine species were not considered in the study of Théry et al. (in press) and it is possible that they correspond to yet two more cryptic species. *Essigella californica* is known to live on at least 34 *Pinus* species (Kimber et al. 2013) and it is likely that other cryptic species await discovery. We are unable to fully evaluate the species complex here due to a lack of material. The taxonomic nature of *Essigella californica* continues to be a complex issue meriting further study. Such a study would require substantial material of representative populations from as many known host plants as possible. A redescription of this species and the members of its complex would require morphometric data and multivariate analyses as per Sorensen (1994), combined with molecular phylogenetic and species delimitation methods as per Théry et al. (in press).

### *Essigella patchae* and *E. pini*

*Essigella pini* is known to be oligophagous on *Pinus* and according to Sorensen (1994), it can be found on pine species of the subgenus Pinus, section Trifoliae, subsection Contortae (notably on *P. virginiana* Miller), subsection Australes (notably on *P. taeda* Linnaeus), and on pine species of the subgenus Strobus, section Quinquefoliae, subsection Strobus (notably on *P. strobus*). It may be also found on species of subsection Sylvestres (Sorensen 1994). The type specimen of *E. pini* was collected in Maryland on *P. virginiana* (Wilson 1919; Sorensen 1994) whereas that of *E. patchae* was collected in Maine on *P. strobus* (Hottes 1957; Sorensen 1994). Genetic material analysed by Théry et al. (in press) came from a Canadian specimen of *E. patchae* collected on *P. strobus* and a US specimen of *E. pini* collected on *P. rigida* (subsection Australes). Our first suspicions are that *E. patchae* could be a more northern species that would feed on pines of subsection Strobus whereas *E. pini* would be more southern developing on pines of both subsections *Australes* and *Contortae*. It could appear curious that Sorensen did not discriminate both species, even though they are morphologically very close. Actually, Sorensen himself collected only species occurring in the western part of USA. Because *E. pini* and *E. patchae* are the only species occurring in the East, all *E. pini* and *E. patchae* specimens that Sorensen studied came from other collections and represented a smaller specimen sample in comparison with other species. Considering the list of specimens Sorensen (1994) studied and those we verified from both USNM and UMSP collections, it is likely that he studied no more than two specimens identified as *E. patchae*, notably the type specimen in poor condition. Those conditions made revelation of significant differences between the two species difficult.

### Molecular data in aphid diagnoses

Aphids represent a relatively well-studied insect group mostly because of their economic importance. Molecular data are most often used in population genetics (Wongsa et al. 2017; Medina et al. 2017). They are used also in works linked with species recognition using barcodes because of their small size and their difficult systematics (Cœur d’acier et al. 2014; Lee et al. 2011). As in other animal groups, new aphid species can be discovered or confirmed using DNA analyses (Depa et al. 2012; Chen et al. 2015; Jiang et al. 2015). The present paper represents the first time that DNA sequence characters have been used in an aphid species diagnosis. Indeed, use of this kind of data and especially substitutions of nucleotides as characters is rare in animal diagnoses (Renner 2016), and rarer in insects. The precedent was established 8 years ago (Brower 2010). The International Code of Zoological Nomenclature does not explicitly recommend DNA sequence data to establish animal taxa, yet nor does it forbid it (ICZN 1999). Other kinds of non-morphological characters are commonly used in other groups. For example, songs or acoustic signals are used to differentiate species in several animal groups and can be considered good diagnostic characters in frogs (Brown and Richards 2008) or in Orthopteran insects (Hertach et al. 2015; Iorgu et al. 2017). In consequence, we judge that the absence, the presence, or the identity of a nucleotide or of a DNA sequence fragment are the molecular equivalent to the absence, the presence, or the shape of a seta, a puncture, or of any other morphological character. We thus support that this kind of DNA character can be used unambiguously in a diagnosis.

## Supplementary Material

XML Treatment for Essigella domenechi

XML Treatment for Essigella gagnonae

XML Treatment for Essigella sorenseni

XML Treatment for Essigella patchae
